# Effects of Aged Polymethylmethacrylate Microplastics on Physiological Biochemistry and Intestinal-Sediment Microbial Communities of *Urechis unicinctus* (von Drasche, 1881)

**DOI:** 10.3390/ani16182829

**Published:** 2026-09-09

**Authors:** Yumeng Huang, Yuting Guo, Wei Feng, Lijuan Gao, Yiran Fu, Jiale Bai, Feng Liu

**Affiliations:** 1 Yantai Institute, China Agricultural University, Yantai 264670, China; 2023505430330@cau.edu.cn (Y.H.); 17539218119@163.com (Y.G.); fengwei851x@cau.edu.cn (W.F.); juanlig@163.com (L.G.); 15673893692@163.com (Y.F.); cau2023505430322@163.com (J.B.); 2 Shandong Key Laboratory of Digital Fisheries, Yantai 264003, China

**Keywords:** bioaccumulation, ecological biomarker, intestine microbe, sediment microbe

## Abstract

Microplastics (MPs) widely distribute in marine environments and generate severe hazards to marine biota, yet little is known regarding the toxic impacts of aged MPs on benthic invertebrates and their surrounding sediment microbiota. This work aimed to fill this research gap by exploring the comprehensive toxicity of aged polymethylmethacrylate microplastics (PMMA-MPs) toward *Urechis unicinctus* and its sediment habitat. A 21-day laboratory exposure trial was performed with two PMMA-MP treatments: environmentally realistic low concentration (10 μg/L) and extreme high concentration (1000 μg/L). Aged PMMA-MPs accumulated in the body wall and intestine of *U. unicinctus*, with markedly higher body wall MPs loads in the high-dose group (*p* < 0.05). High-concentration PMMA-MPs significantly raised intestinal total protein and triggered intense oxidative stress (*p* < 0.01), and the Integrated Biomarker Response value rose dose-dependently. Sediment microbial β-diversity was obviously reshaped (*p* < 0.05), with enriched pathogens including *Klebsiella* and *Enterobacteriaceae* under high MP exposure, while the worm’s intestinal α-diversity and core microbiome remained stable, showing host adaptability. This study reveals multi-level toxic outcomes of aged PMMA-MPs on benthic worms and their sediment microhabitat, offering fundamental data to evaluate the ecological risks of weathered MPs in marine benthic systems.

## Simple Summary

Tiny plastic fragments called microplastics are everywhere in the ocean and harm marine life, but we still know very little about the harmful effects of aged plastic particles on bottom-dwelling sea creatures and the tiny bacteria living in seafloor mud. This research set out to fill this knowledge gap by testing how weathered plastic pieces affect a common marine worm and its muddy living environment. We kept the animals in lab tanks for 21 days with two different plastic levels: a low concentration matching real ocean conditions and an extremely high concentration. The old plastic fragments built up inside the worms’ bodies, especially at the high dose, and triggered physical damage including oxidative stress in their guts. The large amount of plastic also changed the bacterial communities in the surrounding mud and allowed more harmful bacteria to grow, while the tiny living communities inside the animals stayed balanced, showing the animals could adapt to some extent. These findings provide basic evidence to judge the ecological risks of aged plastic waste on seabed ecosystems.

## 1. Introduction

Due to their lightweight, inexpensive and durable properties, plastics are widely used in today’s society [1]. Since the 1950s, global plastic production has surpassed 8.3 billion tons [2]. When released into the environment, plastics undergo physical, chemical and biological degradation, fragmenting into microplastics (MPs, ≤5 mm) and nanoplastics (NPs, ≤1000 nm). Marine ecosystems are the final destination of MPs, as some plastic waste enters the ocean via rivers [3]. MPs have been detected in marine sediments, surface waters and deep-sea environments, indicating extensive contamination of various terrestrial and aquatic habitats [4]. This poses a serious environmental challenge due to their persistence, bioaccumulation and ecological impact. MPs can enter marine organisms through ingestion, filtration, or respiration, and can further be transferred and accumulated across other organisms via complex food webs [5]. Previous studies have focused on typical plastics such as polystyrene (PS), polyethylene (PE) and polyvinyl chloride (PVC) [6].

PMMA is a biocompatible polymer with high mechanical strength, easy processability, deformability, and rapid post-injection curing, which enables broad applications in orthopedic, medical, cosmetic and optical industries [7,8]. Due to massive production and environmental release, PMMA-MPs have been ubiquitously detected in diverse environmental matrices and aquatic organisms [9]. Field investigations from Southeast Asian and Indian coastal sediments reported PMMA-MP abundances ranging from 2 to 38 particles kg^−1^ dry sediment; total microplastic loads in these coastal sediments varied from 120 to 3100 particles kg^−1^ dry sediment, with PMMA accounting for 4–11% of total microplastic particles. For seawater matrices, global near-shore field surveys documented overall microplastic concentrations spanning 0.01–68 μg L^−1^, with most benthic-coastal habitats showing realistic mean levels of 5–15 μg L^−1^ [10]. So, the experimental concentration of 10 μg L^−1^ falls within this environmentally realistic range and represents a field-relevant dose for PMMA-MP exposure. Nevertheless, ecotoxicological data focusing specifically on PMMA-MPs remain limited compared with other widely studied polymer microplastics.

Most plastics undergo a degree of photo-aging. Ultraviolet (UV) radiation strongly contributes to the photo-degradation of plastics in aquatic environments. Upon UV exposure, MPs become brittle and rough-surfaced, with increased specific surface area, mainly due to uneven erosion from photo-oxidation. Studies show that aging gradually transforms PMMA from a smooth, spherical structure into a wrinkled, fragmented morphology. After 14 days, PMMA exhibits enhanced ester oxidation and greater abundance of oxygen-containing functional groups (XPS analysis indicates an O/C ratio rising from 0.2859 to 0.2973), leading to higher surface negative charge density, smaller hydrodynamic diameter, and improved colloidal dispersibility [11]. Due to alterations in their physical and chemical properties, aged MPs may cause more severe harm to organisms [12]. Aged PMMA-MPs have been shown to migrate vertically more effectively in previous studies [13], which will have a more significant impact on benthic ecosystems. All of these consequences indicate that aged PMMA-MPs may cause greater damage to benthic organisms. Hence, this experiment selected aged PMMA for a 21-day exposure study on *Urechis unicinctus* (von Drasche, 1881).

As a key component of marine sediment ecosystems, *U. unicinctus* is widely distributed along the coasts of the Yellow Sea and Bohai Sea [14]. This species contributes substantially to biogeochemical cycling in benthic habitats: its feeding and burrowing behaviors accelerate organic matter decomposition and enhance nitrogen cycling at the sediment–water interface, effectively reducing ammonia and suspended solids in the overlying water. As a filter-feeding organism, it is highly sensitive to microplastic contamination [15]. While existing studies have mainly focused on its adaptation to sulfide and salinity [16], research into the toxicity of aged MPs on *U. unicinctus* and its habitat remains limited.

The aim of this study was therefore to explore the effects of exposure to different concentrations of aged PMMA-MPs on *U. unicinctus*. The specific objectives were to: (1) reveal the species’ physiological responses to MP exposure and the toxic damage induced by varying MPs concentrations; (2) Explore shifts in the intestinal microbiota of *U. unicinctus* and the sediment microbial community under MP exposure; and (3) elucidate the interactive relationship between *U. unicinctus* and its benthic habitat.

## 2. Materials and Methods

### 2.1. Preparation and Surface Physical Characteristics of MPs

The PMMA-MPs were purchased from Yiyuan Bio Co., Ltd. (Shanghai, China). The shape and particle size of the MPs were determined by scanning electron microscopy (SEM) (S-4800, Hitachi, Chiyoda-ku, Japan). To better simulate the morphological characteristics of MPs in the ocean, the plastics were aged under UV light for 15 days. A UVC-ultraviolet lamp (G60T5, Lightbest Co., Ltd., Changzhou, China) was applied for accelerated aging. The vertical distance between the lamp tube and the sample surface was maintained at 15 cm. The homemade ultraviolet (UV) weathering device was composed of four ultraviolet lamps (4 × 60 W UVC bulbs, wavelength at 254 nm). MPs were flipped every 24 h to ensure uniform ultraviolet exposure [17]. It should be noted that 254 nm UVC light does not represent the spectral composition of natural marine sunlight; this accelerated aging protocol was adopted to generate surface-modified MPs in a laboratory setting, rather than to fully reproduce complex in situ marine weathering [18].

Two polymer stock suspensions at 1 g/L were prepared using Milli-Q water. These stock solutions were further diluted to obtain final exposure concentrations of 10 μg/L (environmentally relevant concentration) and 1000 μg/L (representing extreme contamination scenarios such as effluent discharge outlets of wastewater treatment plants) for subsequent exposure experiments. Furthermore, previous experiments mostly used PMMA microspheres, which do not reflect the real presence of PMMA-MPs in the natural environment [19,20]. We used a novel approach involving mechanical crushing to prepare the MPs.

Surfactant Tween-20 was added at a concentration of 0.00001% *v*/*v* to prevent aggregation of MPs and their adhesion to the container walls. Previous studies have shown that this concentration of Tween-20 in experimental water does not exhibit toxic effects on marine invertebrates [21]. The stock suspensions were stored in the dark at 4 °C and sonicated prior to use.

### 2.2. Collection and Pretreatment of Experimental Samples

Several adult specimens of *U. unicinctus* were collected from the sea near Laizhou Bay in Yantai City, Shandong Province, China. Sampling date: 11 May 2024. A total of 270 specimens exhibiting intact external morphology and vigorous activity were selected. *U. unicinctus* samples were then transferred to the laboratory and domesticated in artificial seawater for one week. The seawater used in the experiment was prepared by mixing tap water and sea salt (Beijing, China, Blue Star) in an appropriate ratio (0.036:1). During the acclimatization and the formal exposure experiment, *U. unicinctus* samples were placed in culture tanks and continuously aerated (temperature: 24 ± 2 °C; salinity: 30 ± 1‰; pH: 8.3 ± 0.2). Half of the seawater in the tank was replaced daily and *U. unicinctus* was fed with commercial *Chlorella vulgaris* solution at a concentration of 3 × 10^6^ cells/mL [22]. Feeding was stopped 48 h before the start of the experiment.

Sediments were collected from the sea area near Laizhou Bay in Yantai, China (37°45′ N, 121°51′ E). The sediment was passed through a 40-mesh sieve to obtain the optimal particle size for *U. unicinctus* [23]. The sediment was placed in a −20 °C refrigerator to kill macroorganisms to ensure subsequent determination [24].

A total of 180 intact, active, and domesticated individuals of *U. unicinctus* (weight 25.64 ± 3.74 g; length 12.5 ± 2.3 cm) were selected from the culture tanks and they were randomly divided into three groups. Each group consisted of three replicates, each containing 20 *U. unicinctus.* They were placed in a 40 × 50 × 15 cm aquaculture tank, including a control group (CK), 10 μg/L low-concentration aged PMMA-MPs (L), and 1 g/L high concentration aged PMMA-MPs (H). Each aquaculture tank was covered with 7 cm of sediment [25]. The aquaculture density of *U. unicinctus* was in accordance with the recommendations [26]. MP concentrations used in previous indoor simulation studies are often orders of magnitude higher than those found in nature. To address this issue, we used exposure concentrations of 10 and 1000 μg/L to simulate “environment-related concentrations” in laboratory simulations [27].

During the exposure period, half of the seawater was replaced daily, and MPs were then re-added from the stock suspension at the nominal concentration to maintain the MP concentration in the exposure tank. The exposure period was 21 days, during which no feeding was provided. Additionally, the position of the air stone and aeration intensity were adjusted to prevent excessive stirring and movement of the MPs, ensuring their relatively uniform distribution in the seawater. Visual inspections were conducted to assess the distribution and aggregation of MPs in the water column. MPs were uniformly distributed in the medium without aggregation. Within a short period, the quantity of microplastic particles settling under natural conditions is relatively limited [28].

### 2.3. Examination of MPs Accumulation

Using the experimental protocol published in a 2022 study [29], 18 individuals of *U. unicinctus* were sampled, with 2 individuals per replicate and 3 replicates per group. The mixture of intestinal tissue and coelomic fluid, as well as body wall tissue, were separately digested in 180 mL of 10% KOH solution at 60 °C for 48 h [30]. The digested suspension was subsequently vacuum-filtered using a 1.0 μm glass fiber filter (Whatman GF/B). The filter membrane was transferred into a glass Petri dish and air-dried at room temperature for subsequent microscopic analysis. MPs were identified and enumerated under a stereo microscope (Olympus SZX10, Tokyo, Japan). To eliminate background contamination during sample processing, procedural blanks were established and processed following the identical digestion, filtration and counting procedures as used for tissue samples.

### 2.4. Variation in Body Weight of U. unicinctus

At the beginning of the experiment and on day 21, after allowing the water in the respiratory intestine of *U. unicinctus* to drain naturally [31], 18 individuals of *U. unicinctus* were sampled, with 2 individuals per replicate and 3 replicates per group. The average value per replicate was used for subsequent calculations. Growth performance was evaluated by analyzing weight gain (*WG*, %) and the specific growth rate (*SGR*, %/day) [32], using Equations (1) and (2):

(1) WG=100×(W2−W1)÷W1

(2) SGR=100×(lnW2−lnW1)÷T

*W*1 and *W*2 represent the initial weight (g) and final weight (g) respectively, and *T* is the duration of exposure in days.

### 2.5. Microbial Analysis of the Intestine and Sediment in U. unicinctus

A total of 27 individuals of *U. unicinctus* were sampled, with 3 individuals per replicate and 3 replicates per group. After dissection to collect coelomic fluid, intestinal samples were aseptically removed [31], and the intestinal contents were extruded for subsequent microbiological analysis. Sediment samples were collected from each treatment group using the five-point sampling method to examine the sediment microbial community [33]. Total genomic DNA was extracted from sediment and intestinal tract samples of *U. unicinctus* via a magnetic bead-based DNA extraction kit for sediment and fecal samples, following the manufacturer’s protocols. The V4 hypervariable region of the 16S rRNA gene was amplified with universal primers 515F (5′-GTGCCAGCMGCCGCGGTAA-3′) and 806R (5′-GGACTACHVGGGTWTCTAAT-3′). The PCR program consisted of an initial denaturation at 95 °C for 3 min, 35 cycles of 95 °C for 30 s, 55 °C for 30 s and 72 °C for 45 s, and a final extension at 72 °C for 10 min. Amplicons were checked on a 2% agarose gel, and sequencing libraries were constructed with the NEB Next Ultra™ II FS DNA PCR-free Library Prep Kit (New England Biolabs, Ipswich, MA, USA).

The constructed libraries were quantified using Qubit 4 Fluorometer and qPCR. (https://www.thermofisher.com, accessed 10 August 2026) Qualified libraries were sequenced on the NovaSeq 6000 platform with PE250 reads. Sample data were demultiplexed based on barcode sequences and PCR primer sequences. Raw sequencing data were obtained in FASTQ format. Paired-end reads were preprocessed using Cutadapt v4.4 to detect and remove adapter sequences. After trimming, low-quality sequences were filtered out. Denoising, merging, and chimera removal were performed using DADA2 within QIIME2 (version 2020.11) with default parameters. Finally, the software output an amplicon sequence variant (ASV) abundance table with representative sequences. Representative sequences for each ASV were selected using the QIIME2 package and annotated against the SILVA database (v138).

### 2.6. Measurement of Biochemical Indicators

A total of 18 individuals of *U. unicinctus* (2 individuals per replicate and 3 replicates per group) were selected to measure activities of superoxide dismutase (SOD), catalase (CAT), malondialdehyde (MDA), triglyceride (TG), cholesterol (TC), acid phosphatase (ACP) and alkaline phosphatase (AKP) in the coelomic fluid; Na^2+^/K^2+^-ATPase content and Ca^+^/Mg^+^-ATPase content in the coelomocyte membrane were measured using commercially available assay kits (Nanjing Jiancheng Bioengineering Institute, Nanjing, China). The selection of specific biochemical indicators in *U. unicinctus* for assessing microplastic (MP) toxicity is grounded in their established roles as biomarkers for oxidative stress, lipid metabolism disruption, immune function, and energy metabolism in aquatic organisms. These biomarkers provide a comprehensive suite of insights into the physiological and biochemical impacts of MPs. Unless otherwise specified, all other reagents used in this experiment were of chemical analysis reagent grade. The coelomic fluid sample was placed in a refrigerator at −80 °C for testing.

### 2.7. Integrated Biomarker Response (IBR) Index Analysis

A total of seven representative biomarkers were selected. These biomarkers were selected to represent oxidative stress, immune response, lipid metabolism, and energy metabolism, which are commonly affected by microplastic exposure. Based on the IBR index, a modified approach referenced from the correction method [34] was applied for integration. The calculation procedure is as follows:

The biomarker data (*X*) of each treatment group were compared with the control group data (*X*_0_), and a logarithm was applied to reduce variability, using Equation (3).

(3) Y=log X÷X0

The mean (m) and standard deviation (s) of the biomarkers were calculated, and Z-scores were obtained using Equations (4) and (5).

(4) $$Z=\frac{\left(Y-m\right)}{s}$$

The biomarker deviation index A was defined as:

(5) $$A=Z-Z_{0}$$

where *Z* represents the treatment group and *Z*_0_ denotes the control group.

A star plot was generated, where the magnitude of each biomarker deviation index A in experimental groups is represented by the radial length in the plot. Values above 0 indicate induction of the biomarker, while values below 0 indicate suppression.

The *IBR* value was calculated as Equation (6).

(6) $$IBR=\sum \;\left|A\right|$$

### 2.8. Statistical Analysis

Alpha diversity, based on the Shannon and Chao1 indices, was calculated using QIIME. Principal coordinate analysis (PCoA) was performed using the weighted_unifrac distance metric to visualize sample separation. Column charts showing the relative abundances of the top 10 species were generated using Origin (version 2024). Heatmaps were plotted using R. Linear discriminant analysis (LDA) combined with effect size (LEfSe) was applied to identify characteristic bacteria in each group. Picrust2 performed *t*-tests (*p* < 0.05) on functional pathway differences between different treatment groups based on the KEGG database (https://www.kegg.jp/).

Spearman correlation analysis was employed to assess the correlations among dominant bacteria at the phylum and genus levels. The normality of the remaining data was verified using the Shapiro–Wilk test, while homogeneity of variances was analyzed with Levene’s test. Every measured enzyme indicator is presented as mean ± standard error (SE) and was analyzed using SPSS 27.0 software (SPSS, Chicago, IL, USA). They were compared using one-way analysis of variance (ANOVA) followed by Tukey’s test. When the assumptions were not met, the Kruskal–Wallis test was applied first, followed by multiple comparisons. Statistical significance was defined at *p* < 0.05. Different letters indicate significant differences among treatments. An independent-samples *t*-test was performed to compare the particle size between aged and pristine PMMA microplastics. *p* < 0.05 was regarded as the threshold for statistical significance. All figures were generated using Origin 2024.

## 3. Results

### 3.1. Characterization Changes of Aged Microplastics

**Figure 1:**
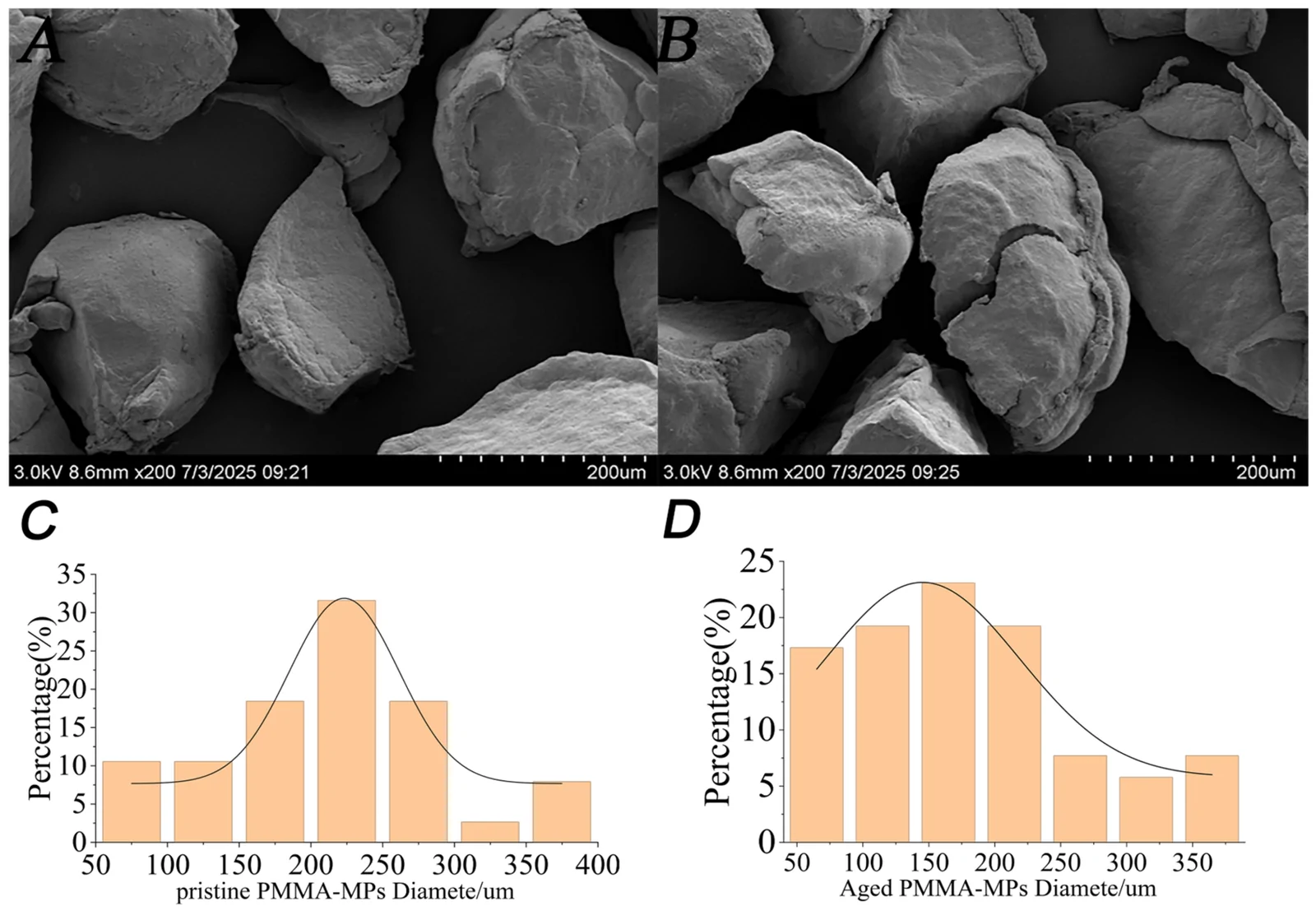
Morphological characterization of PMMA-MPs using scanning electron microscopy (SEM). (**A**) Pristine PMMA-MPs (smooth spherical surface); (**B**) aged PMMA-MPs after 15-day UV irradiation (wrinkled and fragmented surface); (**C**) particle-size distribution of pristine PMMA-MPs; (**D**) particle-size distribution of 15-day UV-aged PMMA-MPs.

Scanning electron microscopy (SEM) (S-4800, Hitachi) was used to observe the surface morphology of pristine and aged PMMA-MPs (Figure 1A,B). Markedly more fissures were observed on the surface of aged MPs relative to pristine MPs. Meanwhile, particle size distribution was quantified based on SEM images via ImageJ 1.54f software to verify the effects of simulated aging treatment. The mean diameter of pristine PMMA-MPs was 216.32 ± 12.25 μm, while that of aged PMMA-MPs was 180.22 ± 12.05 μm (Figure 1C,D). The particle size of aged microplastics showed significant changes relative to pristine microplastics (*p* < 0.05).

### 3.2. MPs Accumulation and Body Weight Changes

**Figure 2:**
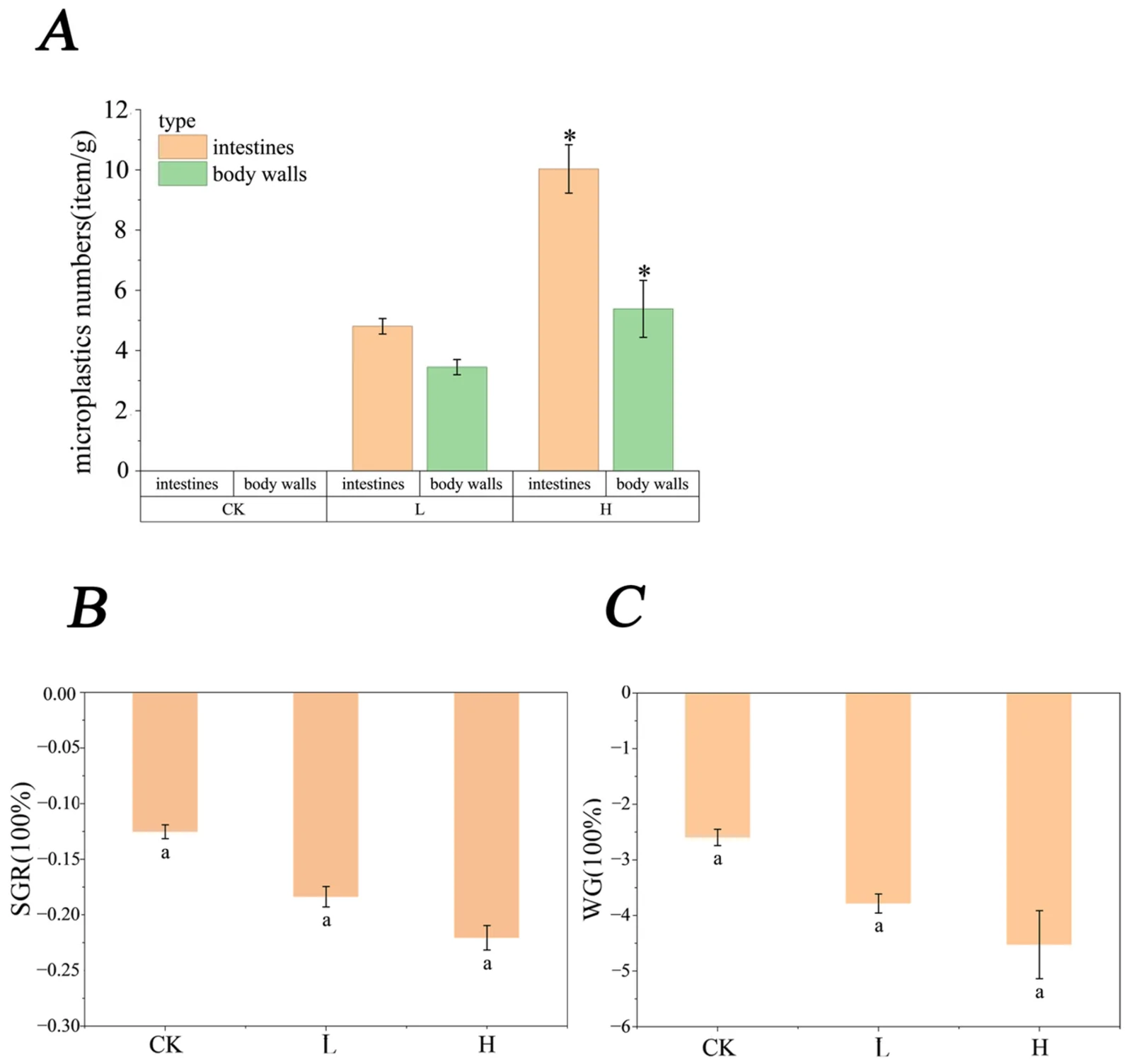
Body weight changes and MP accumulation in *U. unicinctus* under aged PMMA-MP exposure. (**A**) Number of aged PMMA-MP accumulated in the intestine and body wall of *U. unicinctus*. Asterisk (*) indicates a significant difference between group L and group H (*p* < 0.05). (**B**) Growth performance evaluated by specific growth rate (SGR, %/day). (**C**) Growth performance evaluated by weight gain (WG, %). Different lowercase letters above the bars in the same plot indicate significant differences (*n* = 3 per group; one-way ANOVA followed by Tukey’s multiple comparison test; different letters indicate significant differences at *p* < 0.05).

During the filter-feeding process of *U. unicinctus*, enrichment of aged PMMA-MPs was observed simultaneously in both body wall tissue and the intestine. The concentration of MPs in the body wall and intestine of group H was significantly higher than that in group L (*p* < 0.05). Under the same exposure concentration, there was no significant difference in MP intake between the intestine and the body wall (Figure 2A).

After 21 days of exposure, the body mass of *U. unicinctus* in all groups decreased to some extent; however, no significant difference was observed in the mass change between the PMMA-MP-treated groups and the control group (Figure 2B,C).

### 3.3. Total Protein, Oxidative Stress, Immune Defense, Lipid Metabolism, Energy Changes in U. unicinctus Induced by PMMA-MPs Exposure

**Figure 3:**
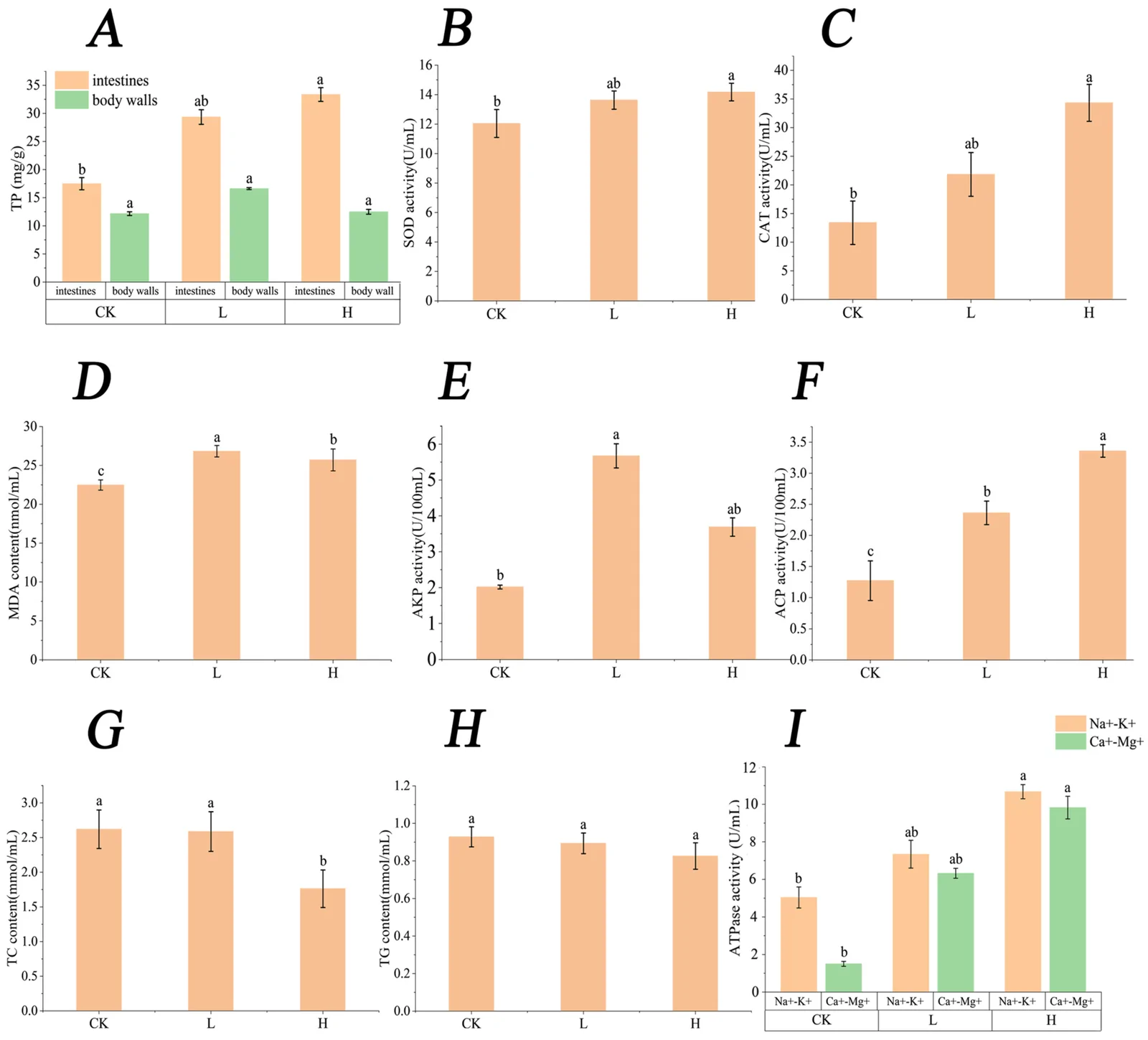
Biochemical indicators were used to reveal the effects of MPs on total protein, oxidative stress, lipid metabolism, and energy metabolism, as well as immunity. (**A**) Total protein (TP) in the intestines and body walls; (**B**–**D**) activities of superoxide dismutase (SOD), catalase (CAT), and malondialdehyde (MDA) in the coelomic fluid; (**E**,**F**) activities of alkaline phosphatase (AKP) and acid phosphatase (ACP) in the coelomic fluid; (**G**,**H**) contents of cholesterol (TC) and triglyceride (TG) in the coelomic fluid; (**I**) contents of Na^+^/K^+^-ATPase and Ca^2+^/Mg^2+^-ATPase in the coelomocyte membrane. Different lowercase letters above the bars in the same plot indicate significant differences (*n* = 3 per group; one-way ANOVA followed by Tukey’s multiple comparison test; different letters indicate significant differences at *p* < 0.05).

**Figure 4:**
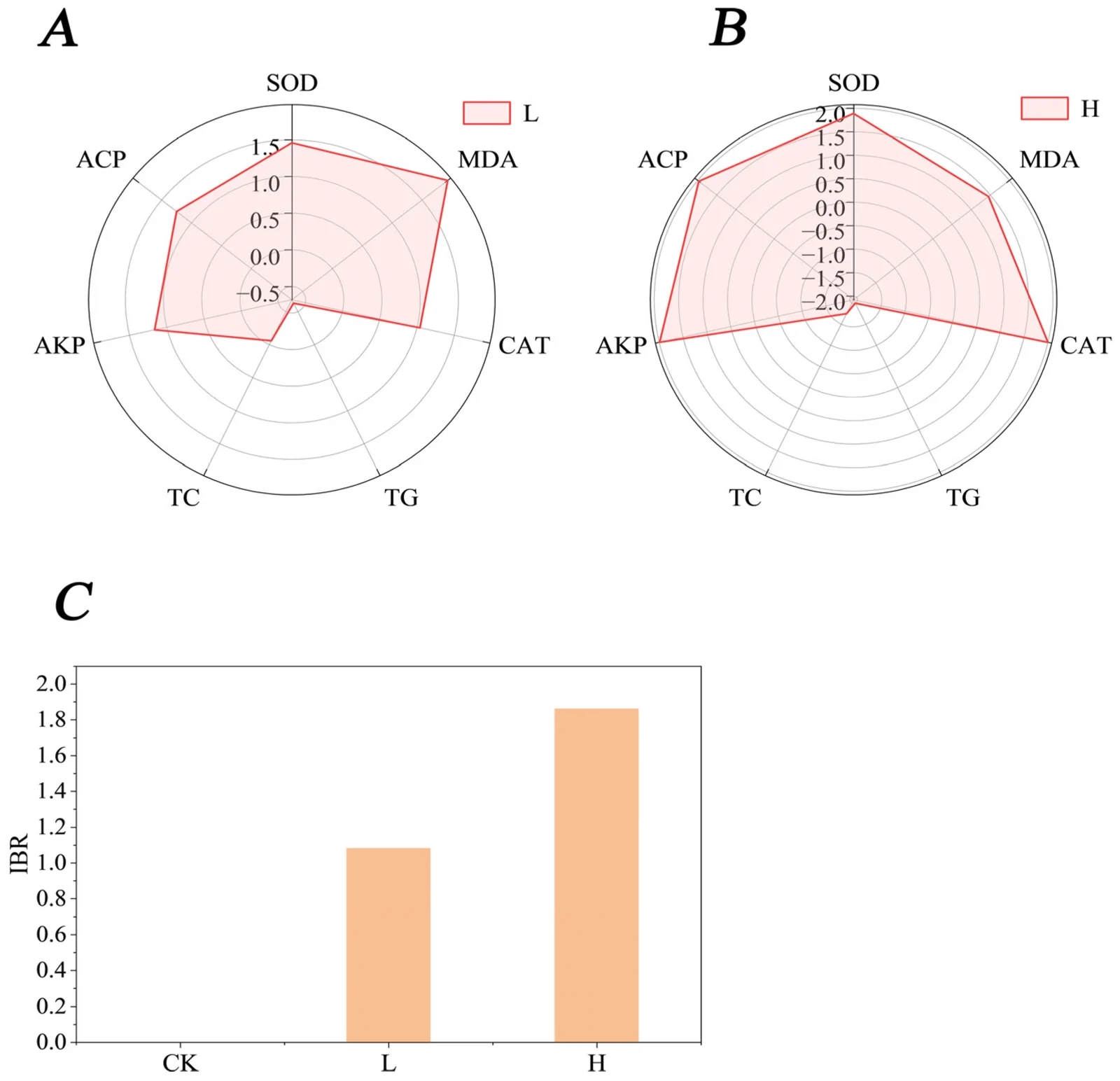
Biomarker star plots and IBR index using seven representative biochemical parameters (measured in the coelomic fluid of *U. unicinctus* after aged PMMA-MP exposure for 21 d. (**A**) Biomarker star plots in L group; (**B**) biomarker star plots in H group; (**C**) IBR index.

The total protein content in the intestines of *U. unicinctus* in group H increased significantly (*p* < 0.05). In contrast, no significant difference was observed in the body wall protein content (Figure 3A). Compared with the control group, SOD activity in the coelomic fluid of *U. unicinctus* was significantly higher in group H (*p* < 0.05). Similarly, CAT activity was significantly elevated in group H (*p* < 0.05). MDA levels increased significantly in both group H (*p* < 0.05) and group L (*p* < 0.05) relative to the control (Figure 3B–D). AKP activity in the coelomic fluid increased significantly (*p* < 0.05) in group L, whereas exposure to high concentrations of aged MPs caused only a slight, non-significant increase. ACP activity increased significantly upon exposure (*p* < 0.05) (Figure 3E,F). Exposure to high-concentration MPs (group H) led to a significant decrease (*p* < 0.05) in total cholesterol (TC) content. In contrast, triglyceride (TG) content showed no significant change during exposure (Figure 3G,H). ATPase content in the coelomocyte membrane of *U. unicinctus* increased significantly in group H (*p* < 0.05) (Figure 3I). After 21 days of exposure to aged PMMA-MPs, transformed data of seven representative biomarkers from the coelomic fluid of *U. unicinctus* were visualized using a star plot. The IBR index of *U. unicinctus* increased with rising concentration of MPs (Figure 4A–C).

### 3.4. Intestinal Microorganisms in U. unicinctus Exposed to Different Concentrations of Aged PMMA-MPs

**Figure 5:**
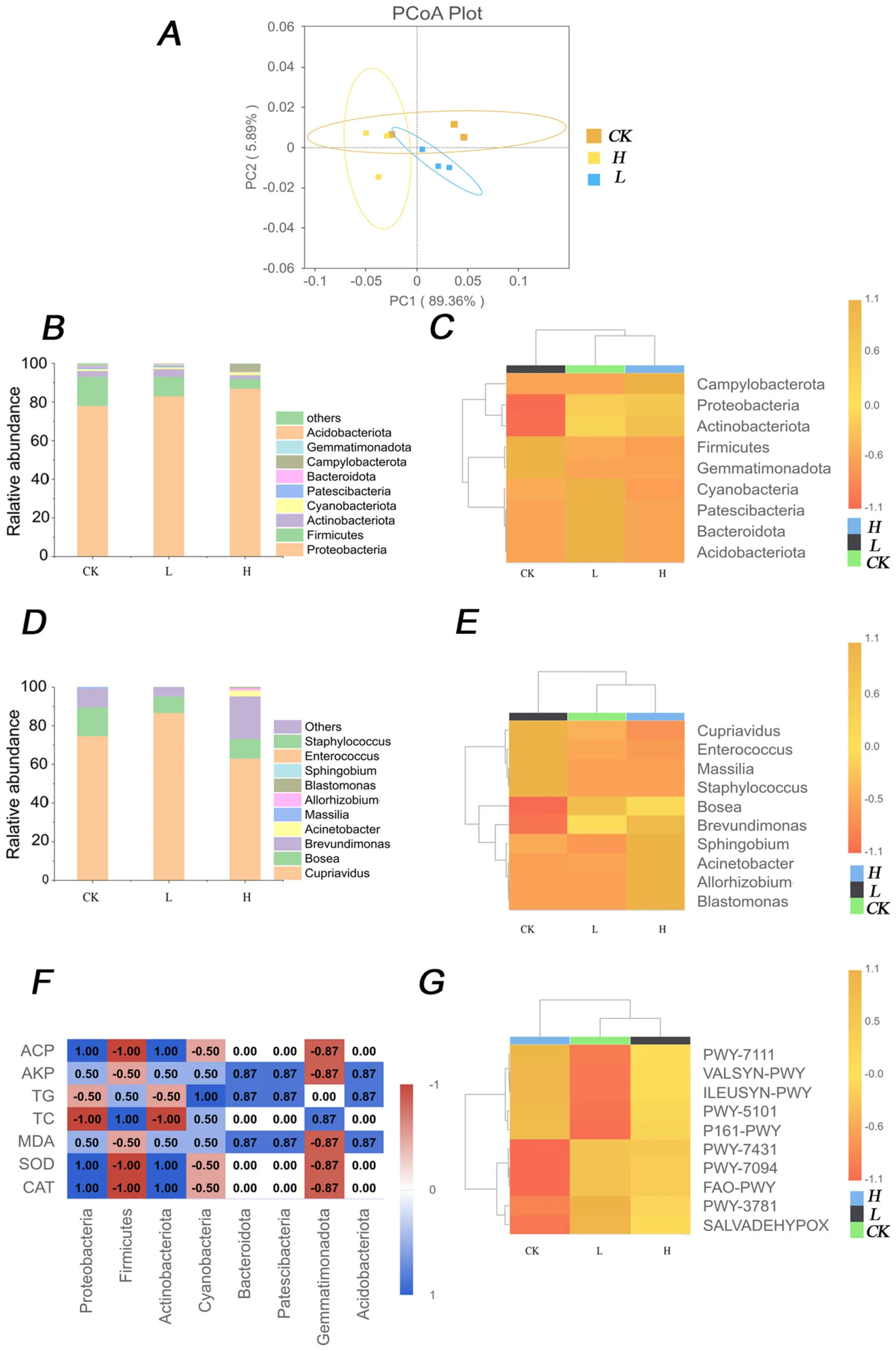
Intestinal microorganisms of *U. unicinctus* in different groups (*n* = 3 per group). (**A**) Principal coordinate analysis (PCoA) based on the weighted UniFrac distance matrix of the intestinal microbiome composition of *U. unicinctus* before and after MPs exposure; (**B**,**C**) intestinal microbiota composition and relative abundance at the phylum level (top 10 phyla); (**D**,**E**) intestinal microbiota composition and relative abundance at the genus level (top 10 genera); (**F**) heatmap of correlation between environmental factors and microorganisms; (**G**) Picrust2 functional abundance clustering heatmap.

The PCoA result indicates that bacterial community structure is influenced by principal coordinate components, which explained 89.36% and 5.89% of the variance, respectively. Analysis of similarities (ANOSIM) verified significant differences (*p* < 0.05) in species composition structure between the aged PMMA-treated group and the control group (CK) (Figure 5A). The microbial species composition (phylum level) in the intestinal of *U. unicinctus* is shown in the figure. *Actinobacteria*, *Proteobacteria*, *Firmicutes*, *Bacteroidetes*, and *Cyanobacteria* are the main bacterial groups in the intestine of *U. unicinctus*. As shown in the figure, under conditions of exposure to aged PMMA-MPs, the dominant species of intestinal microorganisms in *U. unicinctus* underwent few changes. The microbial species composition (genus level) in the intestine of *U. unicinctus* is shown in the figure. At the genus level, the dominant species in the intestinal microbiota of *U. unicinctus* are *Cupriavidus*, *Bosea*, *Brevundimonas*, and *Acinetobacter* (Figure 5B–E). The Chao1 index estimates the actual number of species present in a community by calculating the number of species detected only once or twice. A higher value indicates greater community richness. The Shannon diversity index considers both species richness and evenness. A higher Shannon index value indicates greater community diversity. Compared to the Simpson index, it is more sensitive to community richness and rare species/OTUs/ASVs, making it more suitable for complex communities. Under aged PMMA-MP exposure conditions, the Chao1 index and Shannon index of the intestinal microbiota showed no significant changes (Table S1).

Picrust2 predicts microbial functional profiles and metabolic potential by integrating 16S rRNA gene sequencing data with reference microbial genomes. Figure 5F displays correlation heatmaps between intestinal microbial phyla and host physiological biomarkers of *U. unicinctus*. Figure 5G presents shifts in the relative abundance of predicted microbial metabolic pathways across different aged PMMA exposure treatments. At the phylum level, Proteobacteria exhibited strong significant positive correlations with SOD and CAT (*p* < 0.001) and a significant negative correlation with TC (*p* < 0.001). Firmicutes were significantly negatively correlated with ACP, SOD and CAT (*p* < 0.001), and positively correlated with TC (*p* < 0.001).

### 3.5. Sediment Microorganisms in U. unicinctus Exposed to Different Concentrations of Aged PMMA-MPs

**Figure 6:**
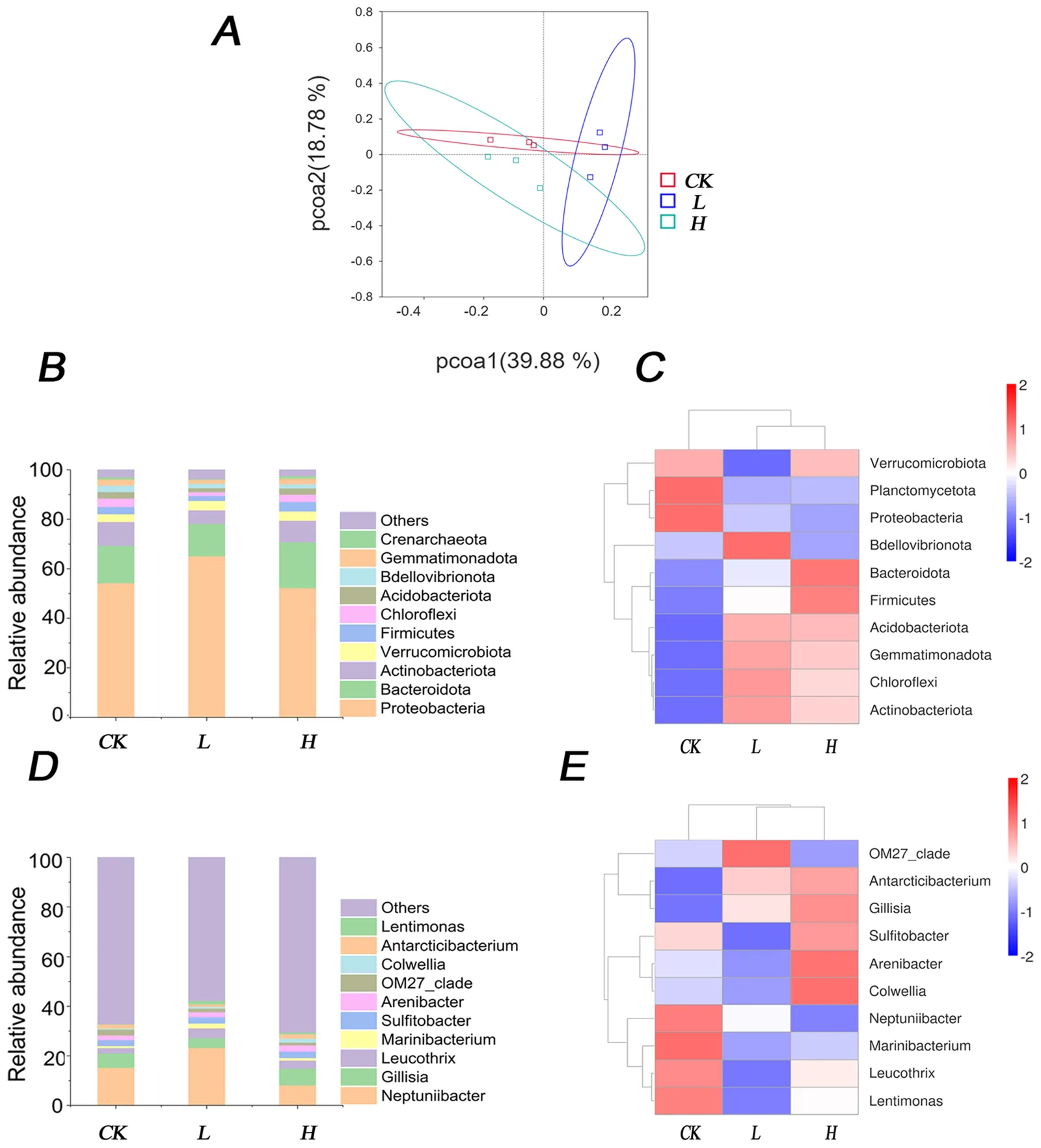
Sediment microorganisms in different groups (*n* = 3 per group). (**A**) Principal coordinate analysis (PCoA) based on the weighted UniFrac distance matrix of sediment microbiome composition before and after aged PMMA-MPs exposure; (**B**,**C**) sediment microbiota composition and relative abundance at the phylum level (top 10 phyla); (**D**,**E**) sediment microbiota composition and relative abundance at the genus level (top 10 genera).

The results showed that bacterial communities were influenced by principal coordinate components PC1 and PC2, by 39.88% and 18.78%, respectively. Visual comparison revealed obvious differences in species composition between the aged PMMA-MP treatment group and the control group (*p* < 0.05) (Figure 6A). α-diversity showed no significant differences between groups (Table S2).

As shown in the figure, the top three bacterial phyla in the sediment are *Proteobacteria*, *Bacteroidota* and *Actinobacteriota.* In the L group, the abundance of the *Proteobacteria* phylum increased from 54.86% to 65.15%, while in the H group, the abundance of the *Proteobacteria* phylum decreased from 54.86% to 52.27%. The top three bacterial genera in the sediment are *Leucothrix*, *Gillisia*, and *Neptuniibacter*. Among these, the abundance of *Neptuniibacter* increased from 15.26% to 23.28% in the L group and decreased to 8.15% in the H group (Figure 6B–E).

**Figure 7:**
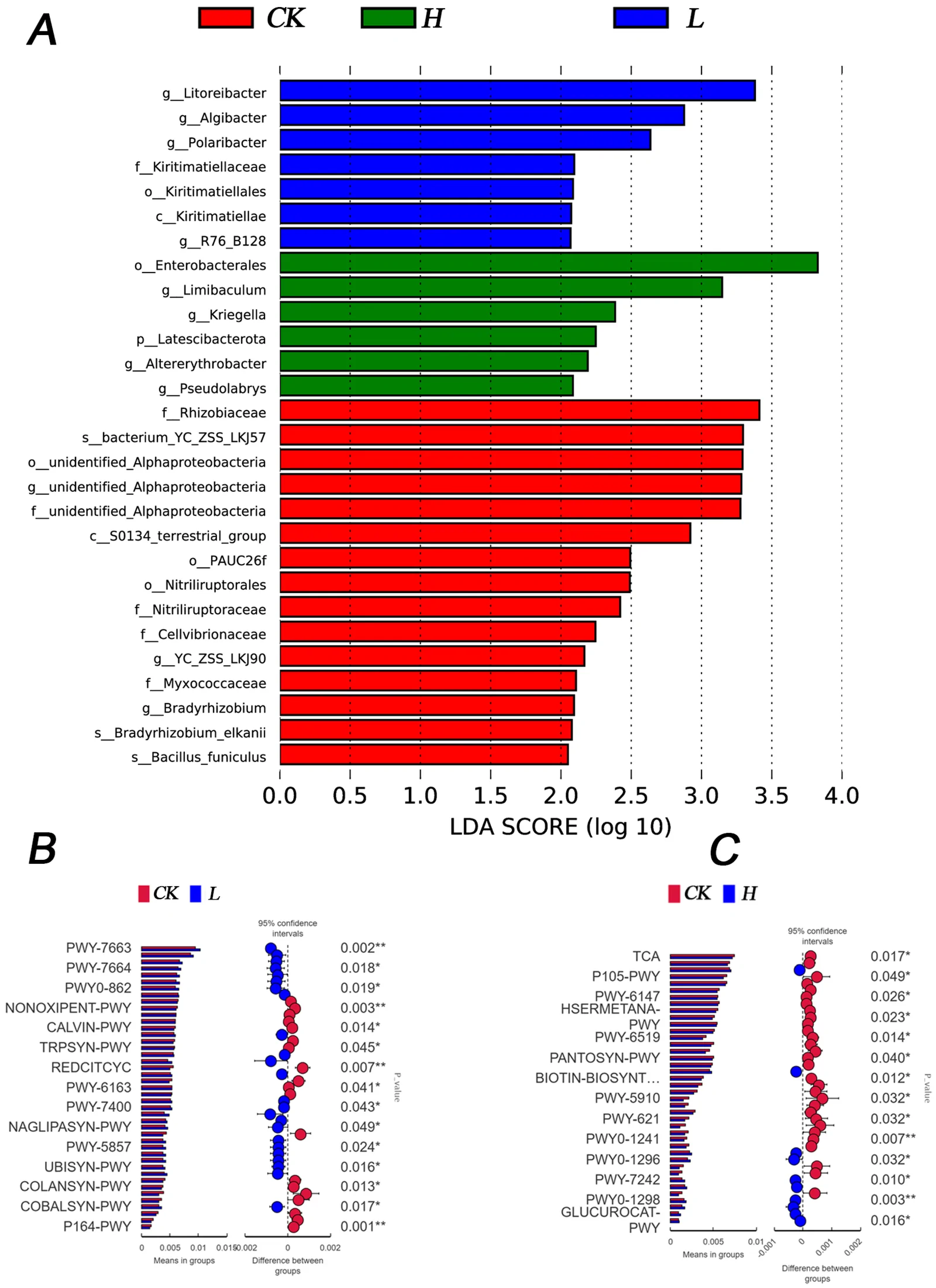
Response of sediment microorganisms to aged PMMA-MP exposure in different groups (*n* = 3 per group). (**A**,**B**) Relative abundance of dominant bacteria screened using the LEfSe analysis (LDA score [log_10_] > 2); (**C**) Tax4Fun-predicted functional abundance clustering heatmap with *t*-test results (* *p* < 0.05, ** *p* < 0.01).

LEfSe Analysis (LDA score [log_10_] > 2) was used to identify the dominant bacteria with relatively lower abundance in each group. The effect of MPs on sediment flora metabolism can be indirectly reflected (Figure 7A,B). Tax4Fun2 is an R package-4.3.1 for predicting microbial functions. Based on 16S rRNA gene sequencing data, it forecasts the functional genes likely present within microbial communities and the metabolic pathways in which they participate. It offers cross-platform compatibility, ease of use, and high memory efficiency. Both treatment groups exposed to two different concentrations of MPs exhibited significant differences in functional pathways compared to the control group (Figure 7C).

## 4. Discussion

### 4.1. Aged PMMA-MP Accumulation and Growth Response in U. unicinctus Analysis of the Accumulation of MPs and Changes in Body Weight

Following 21 days of exposure, the findings indicate that MPs accumulated to varying degrees within the intestine and body wall tissues of *U. unicinctus*. MP accumulation in the H group was significantly greater than in the L group; as with most animals, *U. unicinctus* lacks the necessary enzymes and enzymatic pathways to break down plastics. Consequently, MPs cannot be absorbed or utilized as an energy source [35], leading to persistent MP retention in organs such as the intestine and stomach rather than absorption or utilization as an energy source. This chronic MP accumulation may trigger two interconnected pathways that collectively drive the observed body weight decline across all exposure groups.

MP-induced oxidative stress and immune activation promoted global metabolic disorders, shifting the balance from anabolic growth processes to catabolic energy production [36]. This catabolic state prioritized the breakdown of endogenous protein and lipid reserves to generate ATP for stress repair and vital physiological functions, directly contributing to tissue loss and reduced body mass. Physical and chemical damage to the intestinal epithelium by accumulated MPs impaired the activity of digestive proteases and downregulated the expression of amino acid transporters, resulting in diminished dietary protein absorption and utilization [37]. Unabsorbed proteins accumulated in the intestines, while the resulting energy deficit further exacerbated the breakdown of endogenous protein and fat stores to maintain basal metabolism.

These dual mechanisms—systemic catabolic activation and intestinal nutrient malabsorption—synergistically accounted for the progressive weight loss observed in *U. unicinctus*, although with no significant differences in the rate of decline between exposure groups.

### 4.2. Physiological and Biochemical Mechanisms: Total Protein, Oxidative Stress, Immune Defense, Lipid Metabolism and Energy Changes

Oxidative stress, defined as an imbalance between the production of reactive oxygen species (ROS) and antioxidant defenses, was evident in the high-concentration group. Elevated SOD activity in this group indicates the enhanced conversion of superoxide radicals (O_2_•^−^) to hydrogen peroxide (H_2_O_2_) to mitigate cellular damage [38]. CAT further detoxifies H_2_O_2_ into harmless H_2_O and O_2_ [39], reflecting a coordinated antioxidant response with SOD. In terms of lipid peroxidation, increased MDA content confirms oxidative tissue damage [40]. For immune activation, elevated activities of alkaline phosphatase (AKP) and acid phosphatase (ACP) corroborate this response: AKP is involved in osmoregulation and membrane integrity [41], while ACP is linked to lysosomal defense and phagocytosis [42]. These changes indicate that *U. unicinctus* mounts a robust self-regulatory immune response to MP exposure, consistent with its reported tolerance to foreign contaminants [23].

Collectively, these findings suggest a potential mechanistic hypothesis: MP-induced oxidative stress and immune activation trigger widespread metabolic disturbances marked by inhibited anabolism and enhanced catabolism [43]. This cascade may accelerate endogenous protein and lipid breakdown, which could partially explain the reduced body weight observed in *U. unicinctus*. Concurrently, impaired protease activity and nutrient transporter expression reduce dietary protein absorption, leading to protein retention in the intestinal lumen [28], which may be a key cause of the elevated total protein levels in the intestine. Similar responses have been observed in *M. edulis* and *S. plana* following exposure to 32 μg/L (2 and 6 μm) and 1 mg/L (20 μm) of PS-MPs, respectively [44].

MP exposure disrupts lipid metabolism, as evidenced by reduced total cholesterol (TC) levels, likely driven by lipid peroxidation. This aligns with observations in *Nile tilapia* exposed to nanoplastics, where TC, triglycerides (TGs), and key lipid metabolic enzymes were significantly downregulated [45]. Lipid peroxidation impairs the structure and function of cell membranes, activating inflammatory mediators and further exacerbating metabolic dysfunction.

As mitochondrial stressors, PMMA-MPs affect energy metabolism: elevated ATPase activity in the high-concentration group suggests mild activation of the mitochondrial electron transport system, reflecting an adaptive response to maintain ATP production under stress. This metabolic reprogramming, coupled with impaired nutrient absorption and enhanced catabolism, underlies the observed growth inhibition and body weight loss in *U. unicinctus* [25].

The Integrated Biomarker Response (IBR) has become one of the commonly adopted index models in environmental quality assessment, field biomonitoring, and controlled experiments. The biomarkers currently incorporated are primarily non-specific, including oxidative stress markers, stress proteins, genotoxicity indicators, and lysosomal membrane stability [46]. The IBR index serves as a useful tool for assessing stress responses and ecological risks. A higher IBR value generally indicates increased toxic stress on the tested organisms. After 21 days of exposure to aged PMMA-MPs, transformed data of seven representative biomarkers from the coelomic fluid of *U. unicinctus* were visualized using a star plot. The IBR index of *U. unicinctus* increased with rising concentration, demonstrating that aged PMMA-MPs exert certain toxic effects on this species. Exposure to MPs at high concentrations produced more pronounced toxic effects on *U. unicinctus*.

### 4.3. Analysis of Intestinal Microorganisms in U. unicinctus Exposed to Different Concentrations of Aged PMMA-MPs

Exposure to aged PMMA-MPs for 21 days did not significantly alter the α-diversity of the intestinal microbiota in *U. unicinctus* (Table S1). No significant differences were observed in the Chao1 and Shannon indices among the control, L and H groups (*p* > 0.05), indicating that aged PMMA-MPs exerted limited effects on the overall richness and evenness of the intestinal microbial community.

At the phylum level, the relative abundances of dominant phyla, including *Actinobacteria*, *Proteobacteria*, and *Firmicutes*, remained stable. At the genus level, core genera such as *Cupriavidus*, *Bosea*, and *Brevundimonas* showed no significant fluctuations with increasing MP concentrations. Although PCoA ordination showed apparent separation of bacterial community profiles among treated groups and the control group, PERMANOVA further confirmed significant differences in β-diversity (*p* < 0.05, Figure 5A). Nevertheless, PICRUSt2-based functional prediction suggested that core metabolic pathways such as amino acid biosynthesis and carbohydrate degradation were not markedly perturbed (Figure 5G).

The observed stability of the intestinal microbiota may be partly attributed to the strong homeostasis of *U. unicinctus*. Previous studies have reported that the coelomic fluid of this species exhibits high tolerance to foreign contaminants [16]. Immune enzymes such as ACP and AKP may eliminate harmful microorganisms attached to the surfaces of MPs, thereby reducing potential disturbances to the intestinal microbiota. Furthermore, as a detritivorous species, *U. unicinctus* possesses a stable digestive environment rich in proteases and polysaccharide-degrading enzymes, which may provide a relatively buffered habitat for commensal microorganisms [47].

Notably, some genera, such as *Brevundimonas*, showed a slight increase in relative abundance under MP exposure [48]. Certain strains within this genus have been reported to exhibit plastic-degrading potential, which may contribute to the stabilization of the microbial community. However, direct evidence supporting the degradation of PMMA by these taxa is currently lacking.

### 4.4. Analysis of Sediment Microorganisms in U. unicinctus Exposed to Different Concentrations of Aged PMMA-MPs

PCoA analysis revealed distinct shifts in ASV composition and community structure, indicating that aged PMMA altered the similarity and distribution of sediment microorganisms.

At the phylum level, the relative abundances of *Bacteroidetes* and *Firmicutes* increased in the H group, whereas those of *Acidobacteriota*, *Gemmatimonadota*, *Chloroflexi*, and *Bdellovibrionota* decreased. As *Bdellovibrionota* are predators that regulate microbial turnover and element cycling, their reduced abundance may be associated with the slowing of ecological processes in sediments [47].

The abundance of *Neptuniibacter*, which contributes to marine element cycling, decreased under high-concentration PMMA exposure, implying potential impairment of organic matter decomposition [49]. *Sulfitobacter* and *Arenibacter*, which are involved in hydrocarbon removal and may possess MP-degrading potential [50], increased in the exposed groups. At high concentrations, common pathogenic taxa [51] such as *Enterobacteriaceae* and *Klebsiella* became dominant (Figure 7A), whereas in the L group, taxa related to bioremediation and element cycling (e.g., *Litoreibacter*, *Algibacter*) were more abundant.

Functional analysis further revealed significant perturbations in metabolic pathways. Low-concentration exposure altered pathways related to pyruvate (*p* < 0.01) and polysaccharide (*p* < 0.01) metabolism. High-concentration exposure significantly suppressed the TCA (tricarboxylic acid cycle) (*p* < 0.05) and PWY-5913 (*p* < 0.01) metabolic pathways associated with the tricarboxylic acid cycle, which were significantly different compared to the control group, indicating reduced sediment-cycling capacity. Collectively, these results suggest that sediment microbial communities may exhibit partial adaptation under low concentrations, whereas high concentrations may induce community imbalance.

### 4.5. Comparative Analysis of Changes in Sediment Microbial Community and Intestinal Microbial Community

The present study revealed that the response patterns of intestinal microbial communities in *U. unicinctus* and sediment microbial communities differed significantly after exposure to aged PMMA-MPs. This difference can be mainly attributed to variations in exposure scenarios, environmental buffering capacity, and host regulation.

Sediments act as a major sink for MPs in aquatic environments, where microplastic particles tend to accumulate and persist over long periods, forming a relatively stable, high-dose exposure environment without host-mediated protection [52]. Sustained MP exposure can alter the physical structure, redox status, and resource availability of sediments, thereby directly affecting microbial colonization, nutrient utilization, and metabolic activity [53]. Consequently, both the structure and function of sediment microbial communities changed significantly, indicating their high sensitivity to microplastic exposure.

In contrast, the intestine of *U. unicinctus* provides a tightly regulated, homeostatic microenvironment. Host immune factors may further eliminate harmful microorganisms attached to microplastic surfaces and prevent excessive disturbance to the commensal microbiota. These combined effects maintain high stability in the α-diversity and core microbial taxa of the intestine microbiota.

Symbiotic microbial communities regulated by host physiology and immunity generally exhibit stronger resistance to disturbance, whereas free-living environmental microorganisms are more vulnerable to external perturbations. This difference suggests that sediment microbial communities can serve as more sensitive indicators of microplastic toxicity than host intestine microbiota.

### 4.6. Combined Analysis of Physiological-Biochemical Indicators and Microbial Communities

The integrated analysis of the microbiome and physiological indices revealed that exposure to aged PMMA-MPs can affect oxidative stress and metabolic responses in *U. unicinctus* by altering microbial communities.

In addition to MP accumulation in the intestine, disturbance of the sediment microbial community may indirectly exacerbate host oxidative stress. In the high-concentration treatment, the increased relative abundance of potentially pathogenic bacteria such as *Enterobacteriaceae* and *Klebsiella* in sediments may be associated with elevated production of reactive ROS.

Notably, the intestine microbiota remained highly stable despite systemic oxidative stress in the host. Core intestine microbes including *Brevundimonas*, *Cupriavidus*, and *Bosea* contributed to maintaining intestinal metabolic homeostasis and limiting excessive ROS accumulation. The immune enzymes ACP and AKP inhibited the overgrowth of harmful or opportunistic pathogens, thereby preserving α-diversity and community stability [42].

The present study demonstrated that short-term exposure to aged PMMA-MPs exerted mild effects on the intestine microbiota of *U. unicinctus*, but significantly altered the structure and function of sediment microbial communities. Sediment microorganisms play critical roles in benthic food webs, organic matter decomposition, and biogeochemical cycling. Sustained microplastic pollution may threaten the stability and functional sustainability of coastal marine ecosystems [54]. The higher sensitivity of sediment microbes compared with host-associated microbiota highlights the importance of including environmental microbial indicators in microplastic risk assessment.

## 5. Conclusions

This study systematically reveals for the first time the toxic effects of aged PMMA-MPs on *Urechis unicinctus*. PMMA-MPs accumulate in the organism and induce significant physiological and biochemical damage, while the host exhibits relative tolerance with robust stability of its intestine microbiota, yet the sediment microbial communities in its habitat suffer pronounced concentration-dependent disruption and vulnerable ecological functions, presenting a differential response pattern where the host partially adapts but the habitat is impaired. It provides data support for research on microplastic toxicity in marine benthic organisms, as well as a scientific basis for microplastic risk assessment in marine ecosystems and fisheries resource conservation. However, this study has certain limitations: previous studies have shown that the aging process changes the surface properties, hydrophobicity, and biofilm formation capacity of MPs, thereby significantly affecting microbial attachment, toxicity, and degradation. However, the differential effects between aged and pristine PMMA-MPs were not directly compared in this study, and this issue will be further explored in future experiments; the lack of feeding during the trial may affect the determination of body weight, protein, and energy indicators; constrained by experimental conditions and time, the 21-day exposure duration leaves uncertainty about whether the induced changes in intestine microbiota will persist or worsen under long-term exposure; the applicability of results to other marine benthic animals remains unclear; and the effects of pristine PMMA-MPs on *U. unicinctus* were not investigated.

## Data Availability

The original contributions presented in this study are included in the article/Supplementary Materials. Further inquiries can be directed to the corresponding author.

## Supplementary Materials

The following supporting information can be downloaded at https://www.mdpi.com/article/10.3390/ani16182829/s1. Table S1: Intestinal alpha diversity index; Table S2: Sediment alpha diversity index.
