# Supplementary material for: Effects of Aged Polymethylmethacrylate Microplastics on Physiological Biochemistry and Intestinal-Sediment Microbial Communities of Urechis unicinctus (von Drasche, 1881)
Source: Animals (Basel). 2026 Sep 9;16(18):2829. doi: 10.3390/ani16182829 (PMC13603229; doi:10.3390/ani16182829)
Supplement: Supplementary file 1 [file animals-16-02829-s001.zip › animals-4467900-supplementary.pdf]

Table S1. Intestinal alpha diversity index

Sample_name	shannon	chao1
CK1	1.023	17
CK2	1.271	19
CK3	1.361	18
PMMAL.A1	0.844	19
PMMAL.A2	1.157	13
PMMAL.A3	1.106	14
PMMAH.A1	1.305	12
PMMAH.A2	1.407	16
PMMAH.A3	0.837	16

There was no significant difference between the Shannon index and the Chao1 index (*P<0.05*).

Table S2. Sediment alpha diversity index

Sample_name	shannon	chao1
CK1	7.880508	3105.012
CK2	7.613059	3023.326
CK3	7.539945	2750.507
PMMAL.A1	6.831961	2721
PMMAL.A2	6.268849	2379.045
PMMAL.A3	7.324692	2619.275
PMMAH.A1	7.829805	3194.045
PMMAH.A2	8.102502	3187.34
PMMAH.A3	7.756134	2764.417

. There was no significant difference between the Shannon index and the Chao1 index (*P<0.05*).
